# Coordinate Based Meta-Analysis of Functional Neuroimaging Data; False Discovery Control and Diagnostics

**DOI:** 10.1371/journal.pone.0070143

**Published:** 2013-07-29

**Authors:** Christopher R. Tench, Radu Tanasescu, Dorothee P. Auer, Cris S. Constantinescu

**Affiliations:** 1 Division of Clinical Neurology, University of Nottingham, Queen's Medical Centre, Nottingham, United Kingdom; 2 Department of Neurology, Neurosurgery and Psychiatry, University of Medicine and Pharmacy Carol Davila Bucharest, Colentina Hospital, Bucharest, Romania; 3 Radiological & Imaging Sciences, University of Nottingham, Queen's Medical Centre, Nottingham, United Kingdom; 4 ARUK National Pain Centre, University of Nottingham, Queen's Medical Centre, Nottingham, United Kingdom; West China Hospital of Sichuan University, China

## Abstract

Coordinate based meta-analysis (CBMA) is widely used to find regions of consistent activation across fMRI studies that have been selected for their functional relevance to a given hypothesis. Only reported coordinates (foci), and a model of their spatial uncertainty, are used in the analysis. Results are clusters of foci where multiple studies have reported in the same spatial region, indicating functional relevance. There are several published methods that perform the analysis in a voxel-wise manner, resulting in around 10^5^ statistical tests, and considerable emphasis placed on controlling the risk of type 1 statistical error. Here we address this issue by dramatically reducing the number of tests, and by introducing a new false discovery rate control: the false cluster discovery rate (FCDR). FCDR is particularly interpretable and relevant to the results of CBMA, controlling the type 1 error by limiting the proportion of clusters that are expected under the null hypothesis. We also introduce a data diagnostic scheme to help ensure quality of the analysis, and demonstrate its use in the example studies. We show that we control the false clusters better than the widely used ALE method by performing numerical experiments, and that our clustering scheme results in more complete reporting of structures relevant to the functional task.

## Introduction

Reports of functional neuroimaging studies summarise locations of significant activation or deactivation related to specific tasks. These almost always include coordinates (foci) in Talairach [1] or in Montreal Neurological Institute (MNI) space. Similar studies are often performed independently by different centres, and coordinate based meta-analysis (CBMA) of such studies has been developed with the aim of combining the results and performing statistical inference on them [2]–[12]. The output of these meta-analyses is a set of voxel clusters located where studies commonly report activation. The clusters then indicate which brain structures are involved in the specific task.

Possibly the most widely known of the CBMA methods are the kernel density analysis (KDA) [10] and the activation likelihood estimate (ALE) [13]. The KDA method models spatial uncertainty of each focus by a uniform sphere of specified radius (∼10 mm). The ALE method models the uncertainty with a Gaussian function with full width half max (FWHM) ∼10 mm. KDA seeks clusters of significantly high density of reported foci. The ALE method estimates the probability that there is a focus in any given voxel. The union of probabilities (the ALE) over all reported foci then reflects the probability that there is at least one focus within a voxel, and clusters of significantly high ALE are tested for.

Both KDA and ALE methods have undergone evolutionary development. They have recently shifted towards emphasis on study [8], [9], rather than the individual foci, preventing individual studies having excessive influence. A further development recognises the possible relationship between foci reported within study [5], [9].

Recently the signed differential mapping (SDM) method for CBMA of neuroimaging data has been introduced [11], and incorporates features from both the KDA and ALE methods. This was originally devised for analysis of grey matter changes, and was required to account for both increases and decreases in grey matter density. The method has recently been updated to allow the inclusion of extra statistical parametric maps in the analysis [12]. Much of the focus of this method is on strict inclusion criteria for studies.

The KDA, ALE, and SDM methods perform voxel-wise analysis. While the recent changes to the ALE method have relatively minor impact [8], it has been shown that different approaches to type 1 error control of the high number of statistical tests (testing in every voxel, so ∼10^5^ tests) involved can have a major impact [6]. Originally the ALE method simply specified a conservative level for rejecting the null hypothesis [13]; the SDM method also employs a conservative threshold [11]. Later, control of the false discovery rate (FDR) [14] was imposed [6]. However, control of the FDR may be problematical when the tests are not independent [14], which they are not for this problem [4]. Furthermore, testing of individual voxels has been shown to be inappropriate in voxel-wise fMRI analysis, with tests that consider the topological features of clusters of voxels being preferred [15], [16]. Consequently, an empirical cluster level control has recently been introduced, whereby the size of significant clusters computed under a randomisation of the foci is used; clusters are declared significant only if large compared to those observed under randomisation [4]. The KDA method uses a conservative family wise error rate (FWER) control; this has less power than FDR [17], but fewer false positives. In the updated MKDA method, a cluster size threshold is also imposed, which is similar to that used with the ALE method.

Here we detail a new algorithm for coordinate based meta-analysis of functional neuroimaging studies that: (1) tackles the issue with large numbers of statistical tests and type 1 error control, (2) uses a new clustering algorithm to give more complete reporting of the results, and (3) includes a diagnostic tool to highlight potential problems with the data. Our method is based on the ALE algorithm, which is implemented in the freely available GingerALE (http://brainmap.org/ale) software; but also has some features in common with the KDA method. The most apparent differences are that we truncate the Gaussian functions to contain only 95% of their mass, so they have a reduced sphere of influence similar to the KDA/MKDA method. Furthermore, we perform hypothesis testing only at the reported foci, rather than at each voxel, reducing the number of statistical tests performed (∼10^2^) dramatically. For these reasons we call our method LocalALE. Consequently it is computationally feasible to generate and store many complete experiments under our null hypothesis and analyse the resulting p-values. We can then control the FDR whilst directly taking account of the dependences between the multiple tests. Another new feature of our algorithm is the clustering scheme, which is important both for reporting of the meta-analysis results, and for our new type 1 error control scheme: false cluster discovery rate (FCDR) control. We estimate the expected number of significant clusters within the null generated experiments to control the FCDR, which is directly relevant to the results, and is more interpretable than FDR; it is a similar principle to FDR, but applied to clusters, rather than tests. The use of FCDR in a voxel-wise CBMA would be computationally very intensive, since it would require storage and processing of many (thousands) images. Similar control mechanisms have previously been described for controlling false positive results applicable to functional MRI [15], [16].

## Materials and Methods

### The spatial distribution for reported foci

The *i^th^* focus from study *j* is located at r*_ij_* and has associated with it a spatial distribution.
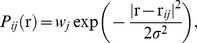
(1)which is Gaussian with standard deviation σ. The weight term *w_j_* normalises the function appropriately, and allows the contribution of each study to be weighted independently, as suggested by [9]. We use



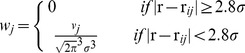
(2)This function truncates the spatial distribution to 95% of its mass (2.8σ), and weights its value by ν*_j_*. One suggested weighting is an appropriately normalised square root of the number of subjects in the study [9]; the studies with larger numbers of subjects are generally considered more robust. However, other factors, such as the significance of the activation or the volume of the activation are also important; unfortunately these are often reported in an inconsistent way. In this study we do not include any weighting factors.

### The Spatial Distribution for a Single Study

This function describes the spatial uncertainty for all foci from a single study.
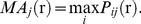
(3)


The *MA* relates only to the nearest focus to point r, and was introduced as the modelled activation (MA) in the ALE method [8].

### The ALE

The ALE is a function of the MA values [5],
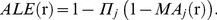
(4)


This is the test statistic we will use in our hypothesis tests. High values indicate a consistent activation across studies.

### Testing for Statistically Significant Clusters of Foci

Pseudo code for the meta-analysis algorithm is given in appendix S1.

It is hypothesised that the studies are related by task/stimulus such that foci are reported more consistently across studies in regions that are important to the task/stimulus. It is these consistently reported regions that are of interest in CBMA, and the ALE is the measure of this consistency; higher ALE values being indicative of more consistent reporting. For hypothesis testing a null distribution of ALE values is needed. The null distribution for CBMA might be obtained by measuring the ALE in experiments using reported foci from studies that are not related by task/stimulus; with the constraints that the number of foci in each study and the number of studies in the experiments are kept the same. However, this may not be practical. By assuming that the foci are independent and uniformly distributed throughout either the grey matter (GM) only as suggested in [4], [9], or throughout the whole brain (WB), a Monte Carlo simulation can be used to computationally generate a null distribution [9]. While this assumption is not strictly valid, it does provide a quantitative scheme for assessing significance in CBMA, and been employed in the various algorithms [9], [13]. However, the distribution of ALE values directly depends on the distribution of MA values. In some studies foci are reported such that they overlap (are separated by a distance <2.8σ) spatially to form clusters, and this affects the MA distribution. In common with the most recent versions of the ALE and MKDA methods, we recognise that within study overlapping foci can form meaningful clusters that should be preserved under randomisation [5], [9]; the aim being to preserve the distribution of MA values.

Our randomisation algorithm is similar to that reported in [9]. Overlapping clusters of foci within the observed studies are identified and for each such cluster (*i*) the centroid (***R*** *_i_*), the mean distance (*d_i_*) of the within-cluster foci from the centroid, and variance of that distance (*S_i_*), are computed. Each cluster centroid is then randomised to a voxel in the mask (GM or WB) with uniform probability. Each focus (*j*) that forms part of cluster *i* is then randomly located at a distance 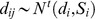 (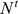 is a truncated Normal distribution such that 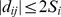 to cut off the long tails of the distribution) from the centroid, in a random direction ***e*** *_ij_* (random vector with uniform probability density on a sphere of radius 1). For this to be a valid randomisation two constraints must be met: 1) the foci should all fall within the mask, and 2) no two centroids (*l* & *m*) must be closer than *d_l_+d_m_+S_i_+S_m_+*2.8σ avoid significant overlapping of the foci when randomised; although with few reported foci per study, overlapping is a relatively rare event even without this constraint. If the randomisation is not valid, it is repeated until the two constraints are met. Again this randomisation of foci is not ideal, but provides a quantitative way of gauging the significances in CBMA that depends directly on the studies included in the analysis.

To compute p-values the ALE value for each reported focus is first computed using equations (3) and (4). Now *ALE*(r*_i_*) is the ALE of focus *i*; note we have now dropped the study index for simplicity. In the *j*th randomisation the *i*th focus is randomised to location v*_ij_* and the ALE value is *ALE*(v*_ij_*). The p-value of the *k*th focus is then
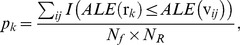
(5)where the sum is over the total number of foci (*N_f_*) in the experiment and the number of randomisations (*N_R_*), and *I*(E) is an indicator function that is 1 if E is true and 0 otherwise.

### Controlling the False Discovery Rate and False Cluster Discovery Rate

Control of type 1 statistical error in neuroimaging is of huge importance, and the adaptive FDR control (BH-FDR) introduced by Benjamini and Hochberg [14] is often employed. The scheme estimates the number of falsely rejected hypotheses from *N* independent tests of level α as α*N*. It then attempts to find the test level where this estimate is at most some small percentage (say 5%) of the number of rejections. There are, however, problems when applied to voxel-wise analysis or fMRI. Firstly the tests in neighbouring voxels are not independent. Secondly, it is not the voxels themselves, but rather clusters of voxels, that should ideally be controlled [15], [16]. Here we detail an FDR method where the numbers of false rejections are estimated directly from many realisations of the experiment generated under the null hypothesis; solving the issue with independence. We then go on to generalise the method to the control of false clusters.

In LocalALE hypothesis tests are performed only at the foci, rather than at every voxel. Consequently, many (*N_E_*) randomised (as described above) experiments can be generated under the null hypothesis computationally feasible requirements; these will be called null experiments to distinguish them from the observed experiment, which consists of the studies included in the meta-analysis. If the *l*th focus in the *m*th null experiment is located at *ρ_lm_*, then its p-value *π_lm_* is estimated by.
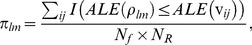
(6)


in exactly the same way as the p-values are estimated for the observed foci. The expected number of false positives for any level α of test, is then
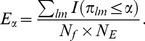
(7)


If the null is rejected *R_α_* times in the observed experiment, then the FDR is controlled at a level γ by maximising α such that *E_α_/R_α_* ≤ γ, with α≤γ. Typically, for example, the FDR is controlled at 5%, so α would be maximised, but at most 0.05, such that the expected number of false positives, *E_α_*, is at most 5% of the total number of rejected hypotheses *R_α_*.

Intuitively, controlling at the cluster level makes considerable sense, as ultimately it is the significant clusters that form the results. In the ALE [4] and MKDA [9] methods, a randomisation procedure is performed and a distribution for the size (number of voxels) of any resulting clusters is generated. The distribution is then used to set a minimum cluster size threshold in the meta-analysis; combined with either FWER or FDR. Here, rather than restricting results to large clusters, FDR is generalised to FCDR control; control of the expected proportion of clusters that are false. The process is exactly analogous to FDR. The p-value is computed for each focus in the null experiments using equation (6) and, for a level α of test, the expected number of falsely significant clusters estimated and compared to the number of clusters declared significant in the observed data; the number of significant clusters is counted using the algorithm detailed in appendix S1. Typically the FCDR is controlled at 5%, so α would be maximised, but at most 0.05, such that the expected number of false clusters is at most 5% of the total number of clusters from the observed experiment.

### Reporting the Results

The results of the meta-analysis are reported in two ways. Equations 1–4 are used to compute an image of ALE values using only the foci declared significant by the analysis. The ALE of the focus with the smallest p-value, but which is not significant, is used to threshold the ALE image. A cluster report is also generated, which consists of the ALE weighted (by the ALE at each focus in the cluster) centroid for each cluster of significant foci and the nearest GM Talairach structure to the centroid, indicating which Talairach structures are important to the task.

### Study Diagnostics: Study Overlap Score

Much of meta-analysis methodology concerns the study inclusion criteria. It is vital to include all studies that test appropriate hypotheses in an appropriate manner. On the other hand it is vital to exclude studies where some methodological problem makes the results in some way inappropriate for the analysis. Furthermore, the act of extracting data for the meta-analysis is often laborious, and can be prone to human error. After a study has met the inclusion criteria, it is prudent to check that it appears commensurate with the other studies. An indication that it is not is useful for diagnostic purposes, and helps pinpoint studies that should be scrutinised further. Data errors can then be fixed, and, if justifiable, studies excluded.

How commensurate each study is with all others is quantified by measuring the overlap of foci between studies. The ALE is computed for each focus within a study, and then averaged. Then each focus within that study is independently randomised, with uniform probability to a voxel in the mask, and the mean ALE recomputed. This is repeated for each study 1000 times, and the proportion of times the mean ALE in the observed study is greater than that under randomisation estimated. A proportion close to one indicates that the study reports foci in similar locations to the other studies. A small proportion indicates overlap that is similar to randomised foci, and that the study should be scrutinised further, and any data extraction errors fixed.

### Experiments

Experiments involving numerically simulated data, and real study data, were performed. Comparisons of results from LocalALE and the latest version of GingerALE [8] were made. We set GingerALE to use its most conservative FDR method [6], controlling at a level of 0.05. Furthermore, we set the lower volume threshold for a significant cluster at 360 mm^3^ (45 voxels, as used in [4]). In LocalALE, we used a WB Talairach mask to generate the null samples (although we also compare with the GM mask), FWHM of the foci was 10 mm, no weighting was applied, and FDR and FCDR were controlled at a level of 0.05. Convergent results were obtained using 10000 permutations for hypothesis testing, and two thousand null studies generated for FDR or FCDR control; increasing these numbers did not change the experiment outcome.

The WB mask is the Colin Talairach image obtained from the brainmap.org website. For the GM mask we performed affine registration of the ICBM 452 T1 structural atlas (http://www.loni.ucla.edu/ICBM/Downloads/Downloads_452T1.shtml) to the Talairach image. The registration parameters were then applied to the GM tissue class image only, and subsequently a threshold applied to the image to leave only the bulk grey matter.

All Coordinates Used were in Talairach Space.

#### Experiment 1

Testing the randomisation of foci. To test our randomisation procedure we extract data from a thermal pain stimulus study (from experiment 5), which reports 71 foci that overlap to form 15 clusters. We calculate the MA for each voxel of the GM mask using equation 2. We obtain the distribution of MA values for the observed data, then randomise the foci as described in the methods section. We then re-compute the distribution of MA values and average over 100 independent randomisations. The two distributions are then compared. We expect the distributions to be similar, on average, if our cluster preserving randomisation algorithm works as required. For comparison we also compute the distribution of MA values for foci randomised independently.

#### Experiment 2

Random experiments. We aimed to examine the frequency of obtaining false clusters using a randomised set of foci as a test of type 1 error control. We used the face perception experiment reported in [4], which has 19 studies looking at brain activation evoked by visually presented faces, and 173 foci; these data were specifically chosen since they allow us to perform cluster level control within GingerALE using the lower cluster size threshold of 45 voxels (360 mm^3^), as reported in [4]. The foci were randomised independently throughout the GM mask, and meta-analysis performed, 1000 times. We estimated the frequency of obtaining false clusters using LocalALE on controlling the FDR, FCDR, and BH-FDR. One hundred of these randomised experiments were also performed using GingerALE for comparison.

#### Experiment 3

Face perception. We repeat the face perception experiment reported in [4]. We examine the diagnostic overlap scores, and perform meta-analysis on the data. Reports of significant clusters found by GingerALE and LocalALE are given. Study data was downloaded from the brainmap database using Sleuth (http://www.brainmap.org/sleuth/) [18]–[20].

#### Experiment 4

Randomising non-significant foci from the face perception experiment. Foci in the face perception data are randomised independently and with uniform probability, except for those that contribute to the significant clusters obtained by LocalALE using FCDR; allowing us to examine the false positive control in the presence of known significant clusters. We then perform meta-analysis on the new set of foci, using GingerALE and LocalALE, to observe if new significant clusters occur out of the randomisation. We expect the original clusters to be present still and few new clusters if the false positives are well controlled. We run the experiment 1000 times using LocalALE, and 100 times using GingerALE, and produce a histogram of the number of extra significant clusters detected.

#### Experiment 5

Meta-analysis of thermal pain stimulation in healthy volunteers. To compare the algorithms in a larger experiment, we perform meta-analysis of thermal pain stimulation, which has been widely studied using fMRI. Extensive functional activation is observed with pain stimulus, so this experiment allows us to test our clustering scheme, which makes use of Dijkstra's shortest path algorithm [21], given in appendix S1.

A search for fMRI studies of experimental thermal-induced pain in healthy people was performed through standard literature databases (ScienceDirect, and PubMed). We used the keywords fMRI and thermal or pain. The references of these articles were then assessed for additional studies that could be considered for inclusion. We excluded single-subject reports, studies using a-priori region of interest (ROI) based analyses, and studies that reported only a restricted field of view. Only activation foci were included. Thirty eight articles were retained, with a total of 49 experiments including 616 subjects and 816 foci. If the pain stimulus was applied on the left, reported foci were reflected about x = 0. Reports of significant clusters found by GingerALE and LocalALE are given. We also examine the diagnostic overlap scores for this data.

To test the sensitivity of FCDR to the clustering algorithm, we also analyse the pain data using a simplified clustering scheme; we used the same algorithm detailed in appendix S1 except for the constraints on the ALE, which were removed.

## Results

### Testing the Randomisation Algorithm

**Figure 1:**
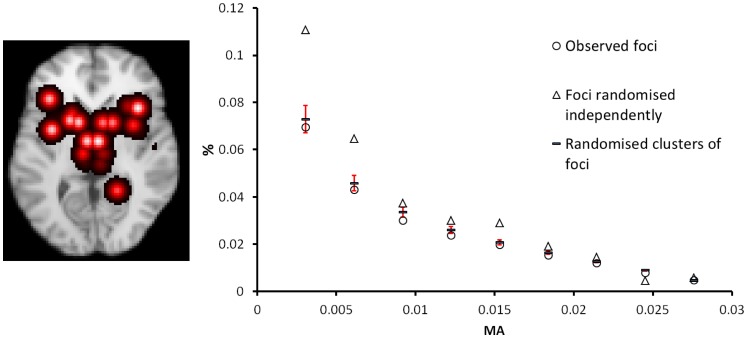
The MA values (red overlay) show the clustering of foci reported within a single experiment; 15 clusters and 71 foci. A scatter plot showing the distribution of (non-zero only) MA values for this experiment depicts: the original MA distribution (circle marker), the MA distribution on randomisation of the clusters (- marker) with error bars (standard deviation), and the distribution after independent randomisation of the foci (triangle marker). The randomisation of the clusters preserves, on average, the observed distribution as required. The distribution of the MA values on randomising the foci independently has a higher frequency of low MA values as expected.

Figure 1 depicts the MA values (overlaid) and clustering within study is clearly evident. The distribution of MA values (>0 only) is shown on the histogram, along with the distribution when the foci have been randomised. When the foci are randomised to preserve within experiment clusters, as described in the methods section, the MA distribution is conserved on average. Randomising the foci independently and with uniform probability through the mask increases the frequency of low MA values as the foci spread out more. The percentage of voxels with zero MA (not shown) is 76% for the observed foci, a similar 77% on average when randomised to preserve clusters, and a lower 68% on average when randomised independently. Without preserving the clustering, the distribution of MA values would result in increased ALE values, and result in a more conservative method.

### Random experiments

**Figure 2:**
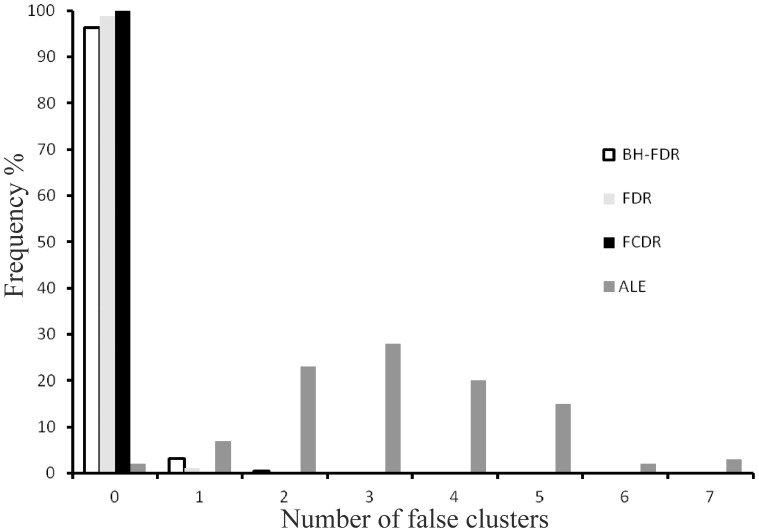
The random foci experiment, involving randomisations of the face perception experiment. Shown are the numbers of clusters found for each random experiment. This experiment examines the frequency of false cluster discovery in the absence significant clustering.

Figure 2 reports the number of significant clusters on analysing the randomised foci experiments using LocalALE and GingerALE. No significant clusters were reported in any of the 1000 experiments using FCDR, while BH-FDR and FDR found significant clusters in only a small percentage of experiments. GingerALE, on the other hand, reports a median of 3 significant clusters per experiment, with an average size of 575 mm^3^ (72 voxels).

### Face perception

**Figure 3:**
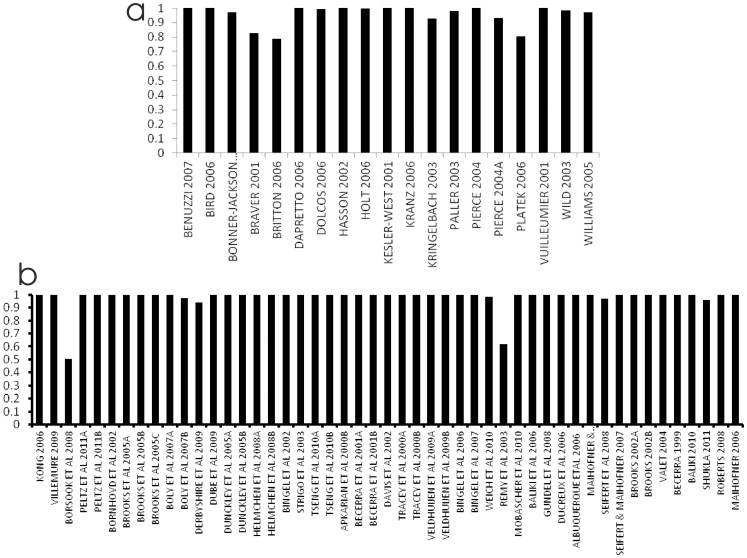
Overlap measures for face perception experiment (a), and the pain stimulus experiment (b).

Figure 3a shows the results of the overlap measure, used for diagnostic purposes. While many of the studies overlap with values close to 1, there are outliers. Platek ’06 [22] has results recorded as MNI coordintates in the brainmap database, but they are actually Talairach coordinates; on correcting this, the overlap increased from 0.80 to 0.94. Braver ‘01 [23] was designed to study working and long term memory tasks only in the prefrontal cortex; inclusion of this study is possibly inappropriate since it does not consider the whole brain, unlike the other studies in the analysis. Other apparent outliers are most likely due to experimental design subtlety, or because the overlap estimate is noisy where few foci are reported.

**Figure 4:**
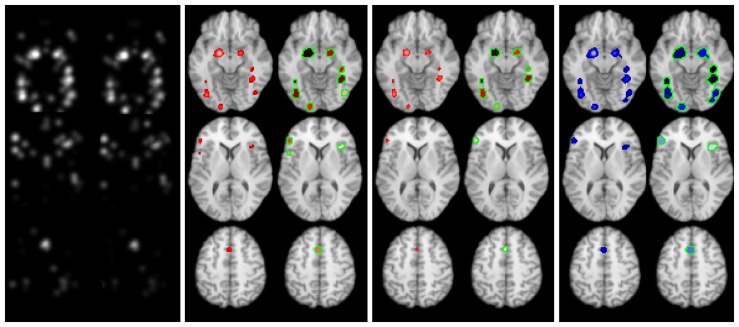
ALE images and statistically significant clusters found on meta-analysis of the face perception data using LocalALE (red) and GingerALE (blue). In column 1 the left images are ALE values computed using LocalALE, and the right ALE values from GingerALE. Column 2 shows results using FDR control, and column 3 FCDR control. In columns 2–4, the left images show the ALE computed using only significant foci, while the results of the respective clustering algorithms are shown on the right.

Figure 4 shows the clusters found using FDR and FCDR control (LocalALE) and FDR control (GingerALE) at a level of 0.05; the Talairach regions involved are reported in table 1. There is little difference using the WB or the GM mask in LocalALE. LocalALE controlled by FDR produced extra clusters that were not found by GingerALE, and vice-versa. These discrepant clusters were either quite small (GingerALE) or only just significant and involving few experiments (LocalALE). FCDR control resulted in fewest clusters, and did not find any of the discrepant clusters.

**Table 1:** Face perception results; significant results from GingerALE compared to those in LocalALE.

Structure	Talairach x, y, z	Studies	GingerALE cluster vol. (mm^3^)	LocalALE GM mask		LocalALE WB mask	
				FDR	FCDR	FDR	FCDR
Left Cerebrum, Amygdala	−18.1, −7.4, −8.4	8	2544	0.0051*	0.014*	0.0052*	0.013*
Right Cerebrum, Amygdala	18.6, −6.5, −10.9	5	1456	0.0053*	0.014*	0.00055*	0.013*
Left Cerebrum, Fusiform Gyrus, BA 37	−38.8, −48.7, −16.7	9	2648	0.0051*	0.014*	0.0052*	0.013*
Right Cerebrum, Fusiform Gyrus, BA 37	36.9, −49.4, −15.0	8	3504^M^	0.0051*	0.014*	0.0052*	0.013*
Right Cerebrum, Parahippocampal Gyrus, BA 36	35.4, −34.8, −13.8	4	3504^M^	0.0059*	0.015*	0.0062*	0.015*
Left Cerebrum, Fusiform Gyrus, BA 19	−37.5, −72.5, −12.9	7	2320	0.0051*	0.014*	0.0052*	0.013*
Right Cerebrum, Fusiform Gyrus, BA 19	39.6, −71.1, −9.6	6	1744	0.040*	0.081	0.067	0.13
Left Cerebrum, Lingual Gyrus, BA 17	−14.2, -93.6, −10.4	3	760	0.0064*	0.015*	0.0068*	0.015*
Left Cerebrum, Inferior Frontal Gyrus, BA 45	−48.7, 29.2, 6.0	3	656	0.0085*	0.021*	0.0090*	0.021*
Left Cerebrum, Medial Frontal Gyrus, BA 32	−0.7, 9.6, 47.4	3	696	0.015*	0.015*	0.015*	0.032*
Left Cerebrum, Precentral Gyrus, BA 44	−48.7, 9.2, 8.8	2	−	0.040*	0.080	0.043*	0.095
Right Cerebrum, Insula, BA 13	34.4, 19.2, 7.4	3	440	0.042*	0.084	0.045*	0.091
Left Cerebellum, Declive	−22.1, −78.1, −16.8	3	−	0.046*	0.09	0.049*	0.094
Right Cerebrum,. Superior Parietal, Lobule. BA 7	28.0, −55.2, 40.2	−	736	0.071	0.12	0.072	0.13
Right Cerebrum,.Middle Frontal Gyru,.BA 46	42.4, 38.3, 20.9	−	384	−	−	−	−
Right Cerebrum,.Inferior Frontal Gyrus,.BA 45	47.0, 25.4, 17.5	−	456	−	−	−	−

LocalALE results are given as estimated FDR and FCDR rates for each significant cluster found. LocalALE results obtained using the whole brain (WB) and grey matter masks (GM) are given for comparison. Significant results in LocalALE are indicated by *. A ‘–’ indicates that that the cluster is not found. Cluster volume is as reported by GingerALE, and merged clusters are indicated by superscript M.

**Figure 5:**
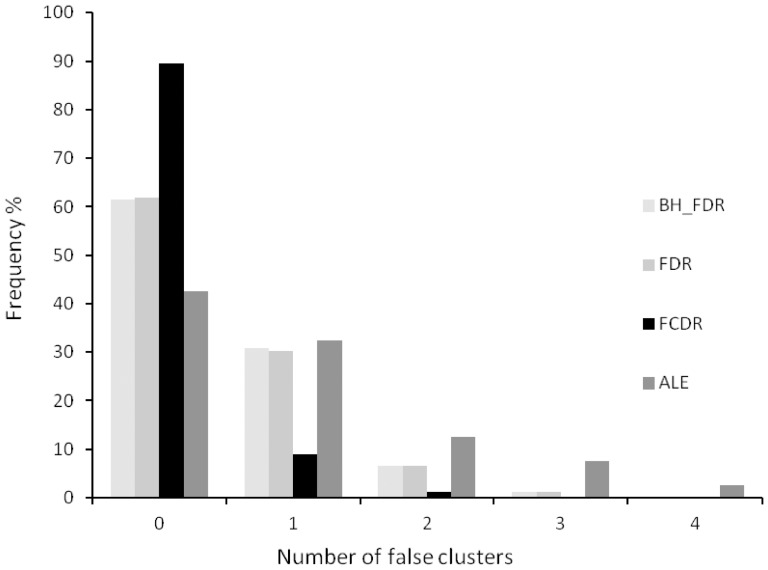
Number of false clusters arising from randomisation of the non-significant foci only in the pain perception data; foci involved in statistically significant clusters found by LocalALE (FCDR) are not randomised. This experiment examines the frequency of false cluster discovery in the presence of known significant clustering.

Figure 5 shows the frequency of finding false clusters when the significant foci, by FCDR, are kept while the other foci are randomised independently and with uniform probability throughout the mask. All methods detect the preserved clusters. FCDR controlled the false clusters best here.

### Thermal Pain Stimulus Data

Figure 3b shows the results of the overlap measure, used for diagnostic purposes. While many of the studies overlap with values close to 1, there are outliers. Borsook et al. (overlap score 0.53) used MNI space for analysis, but coordinates were subsequently adjusted to an MRI atlas of the human cerebellum. Remy et al. (overlap score 0.61) performed an experiment to study how pain modulates brain activity during the performance of a semantic cognitive task; while the paper reports pain as a main effect, on closer scrutiny the experiment was never performed with painful stimulus in isolation of the cognitive task. Both of these studies were excluded from further analysis.

**Figure 6:**
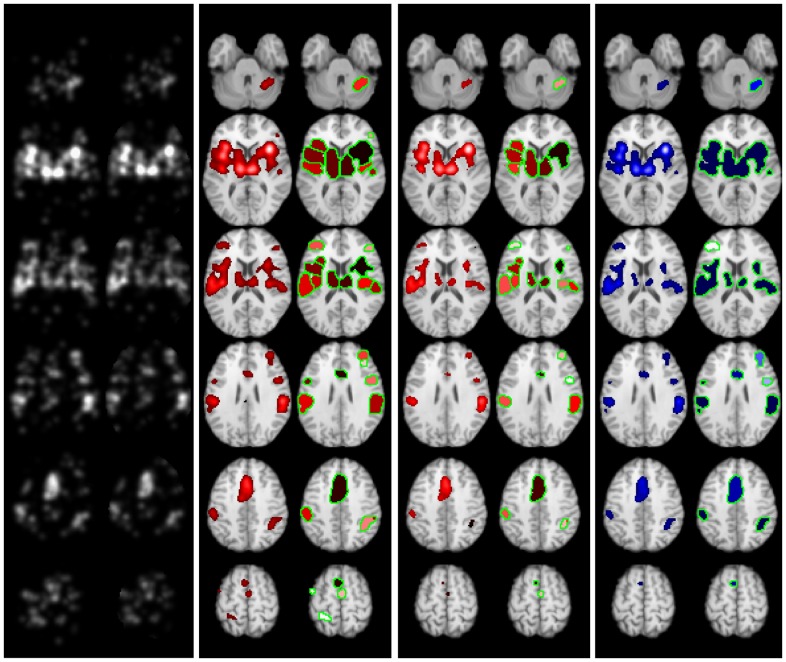
ALE images and statistically significant clusters found on meta-analysis of the thermal pain stimulus data using LocalALE (red) and GingerALE (blue). In column 1 the left images are the ALE values computed using LocalALE, and the right ALE values from GingerALE. Column 2 shows results using FDR control, and column 3 FCDR control. In columns 2–4, the left images show the ALE computed using only significant foci, while the results of the respective clustering algorithms are shown on the right.

Figure 6 shows the clusters found using FDR and FCDR control (LocalALE) and FDR control (GingerALE) at a level of 0.05; the Talairach regions involved are reported in table 2. There is extensive clustering with this dataset, and many anatomical structures involved (table 3). From figure 6 it is clear there are distinct regions with high ALE values where the density of experiments reporting foci is high. Our new clustering algorithm is able to detect these regions, while GingerALE merges multiple clusters. Consequently the cluster report from LocalALE is most complete and informative.

**Table 2:** Studies included in the thermal pain stimulus meta-analysis.

Experiments included: author, year	No subjects	No of Foci
KONG ET AL 2006	16	13
VILLEMURE ET AL 2009	14	19
BORSOOK ET AL 2008	12	6
PELTZ ET AL 2011A/B	11	22/18
BORNHOVD ET AL 2002	9	18
BROOKS ET AL 2005A/B/C	14	15/13/8
BOLY ET AL 2007A/B	24	20/7
DERBYSHIRE ET AL 2009	12	6
DUBE ET AL 2009	12	43
DUNCKLEY ET AL 2005A/B	10	19/8
HELMCHEN ET AL 2008A/B	14	5/71
BINGEL ET AL 2002	14	12
STRIGO ET AL 2003	7	28
TSENG ET AL 2010A/B	12	25/31
APKARIAN ET AL 2000	7	3
BECERRA ET AL 2001A/B	8	31/50
DAVIS ET AL 2002	7	19
TRACEY ET AL 2000A	6	13/12
VELDHUIJEN ET AL 2009A/B	10	10/10
BINGEL ET AL 2006	19	20
BINGEL ET AL 2007	20	21
WEICH ET AL 2010	16	25
REMY ET AL 2003	12	7
MOBASCHER ET AL 2010	32	17
BALIKI ET AL 2006	11	16
GUNDEL ET AL 2008	13	13
DUCREUX ET AL 2006	6	25
ALBUQUERQUE ETAL 2006	8	10
MAIHOFNER & HANDWERKER 2005	12	11
SEIFERT ET AL 2008	14	6
SEIFERT & MAIHOFNER 2007	12	19
BROOKS 2002A/B	18	12/11
VALET 2004	7	18
BECERRA 1999	6	16
BALIKI 2010	16	17
SHUKLA 2011	10	12
ROBERTS 2008	10	17
MAIHOFNER 2006	14	18

**Table 3:** Pain stimulus results; significant results from GingerALE compared to those in LocalALE.

Structure	Talairach x, y, z	Studies	Ginger ALE Cluster volume (mm^3^)	LocalALE WB mask	
				FDR	FCDR
Right Cerebrum, Claustrum	31.5, 10.7, 4.7	37	70528^M^	0.0021*	0.009*
Right Cerebrum, Thalamus, Medial Dorsal Nucleus	8.2, −15.2, 5.9	29	70528^M^	0.0021*	0.009*
Left Cerebrum, Thalamus, Ventral Lateral Nucleus	−10.0, −13.4, 4.8	31	70528^M^	0.0021*	0.009*
Left Cerebrum, Insula, BA 13	−38.2, 4.0, 8.1	33	70528^M^	0.0021*	0.009*
Left Cerebrum, Claustrum	−33.2, 15.7, 2.6	29	70528^M^	0.0021*	0.009*
Right Cerebrum, Inferior Parietal Lobule, BA 40	53.4, −29.5, 23.6	31	70528^M^	0.0021*	0.009*
Left Cerebrum, Insula, BA 13	−37.8, −14.8, 11.3	23	70528^M^	0.0021*	0.009*
Left Cerebrum, Inferior Parietal Lobule, BA 40	−52.3, −26.1, 23.9	33	70528^M^	0.0021*	0.009*
Right Cerebrum, Insula, BA 13	36.3, −16.8, 12.9	17	70528^M^	0.0021*	0.009*
Right Cerebrum, Inferior Parietal Lobule, BA 40	37.7, −48.5, 43.2	14	70528^M^	0.0064*	0.065
Right Cerebrum, Inferior Frontal Gyrus, BA 46	43.6, 36.7, 11.5	9	70528^M^	0.0075*	0.075
Left Cerebrum, Cingulate Gyrus, BA 32	−1.3, 9.5, 39.0	38	12896	0.0021*	0.009*
Right Cerebellum, Cerebellar Tonsil	30.7, −51.0, −30.6	14	2192	0.0021*	0.009*
Right Cerebrum, Middle Frontal Gyrus, BA 10	34.2, 43.9, 24.4	10	1632	0.0043*	0.039*
Right Cerebrum, Middle Frontal Gyrus, BA 9	36.6, 29.8, 30.7	10		0.016*	0.16
Right Cerebrum, Inferior Frontal Gyrus, BA 9	47.7, 4.1, 26.8	13	1600	0.0044*	0.039*
Left Cerebrum, Middle Frontal Gyrus, BA 10	−36.1, 39.5, 15.8	15	1224	0.0044*	0.039*
Left Cerebrum, Medial Frontal Gyrus, BA 6	−0.2, −10.3, 56.0	8	−	0.0092*	0.90
Left Cerebrum, Precentral Gyrus, BA 6	−39.8, −7.9, 54.1	6	−	0.030*	0.31
Left Cerebrum,Superior Parietal Lobule, BA 7	−23.5, −45.7, 60.3	7	−	0.035*	0.35

LocalALE results are given as estimated FDR and FCDR rates for each significant cluster found. Significant results in LocalALE are indicated by *. A ‘–’ indicates that that the cluster is not found. Cluster volume is as reported by GingerALE, and merged clusters are indicated by superscript M.

**Figure 7:**
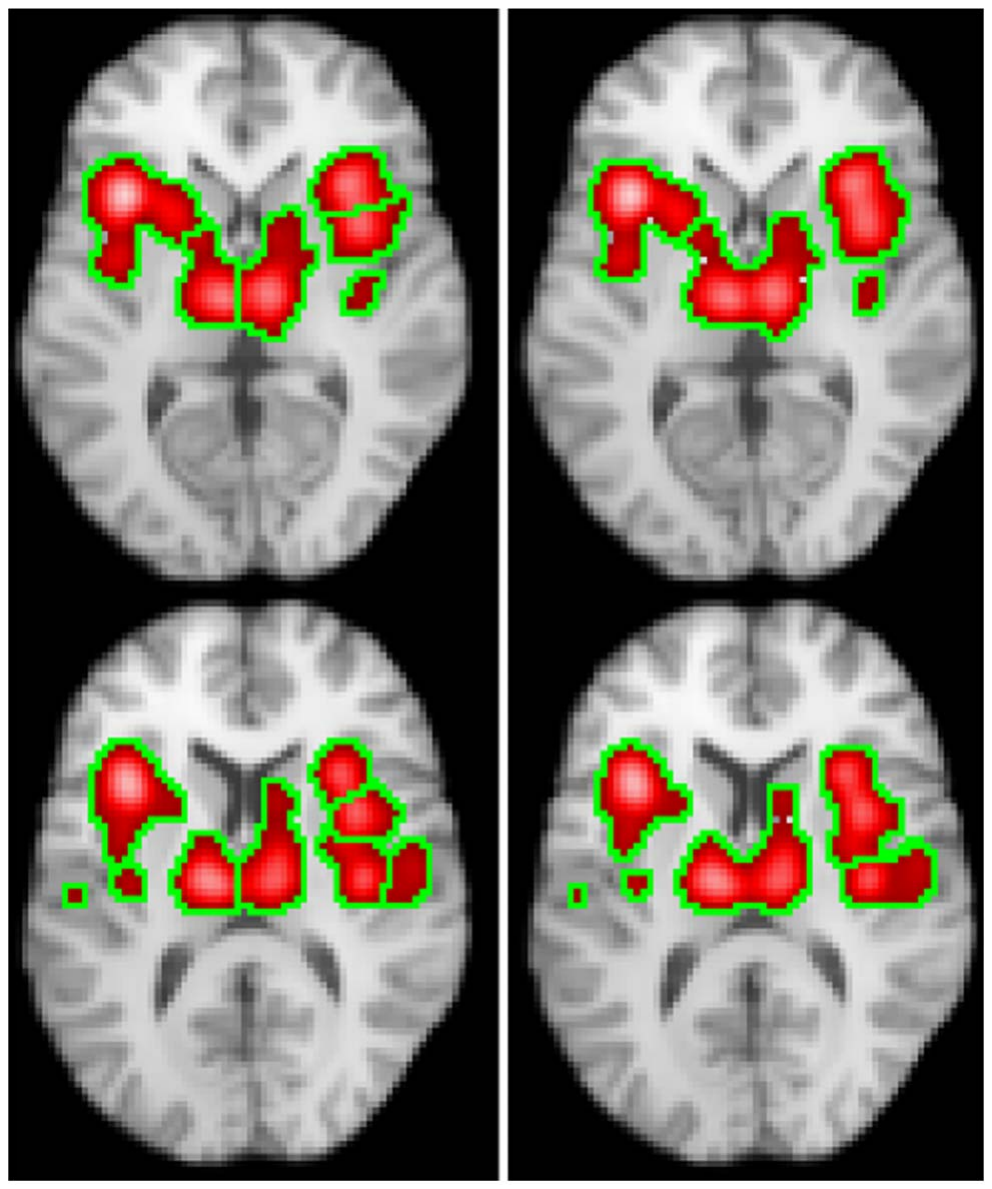
Clusters obtained with the pain data using the clustering algorithm described in appendix S1 (left), and also using a simplified algorithm that ignores the ALE (right). While the clustering is different, the significant regions are very similar.

Figure 7 shows how modifying the clustering algorithm such that it is independent of the ALE values at each focus modifies the clusters. Nevertheless, the FCDR algorithm has still given very similar results. This suggests that FCDR is quite robust to the specifics of the clustering scheme used.

## Discussion

We have developed a new algorithm for performing coordinate based meta-analysis of fMRI studies that have a particular task type in common. The results are clusters located within brain structures that are important to the task. We have tackled one of the major issues with previously reported CBMA methods: type 1 statistical error control. To achieve this it was necessary to develop a new clustering algorithm, which allows clusters to be counted appropriately. The clustering algorithm also produces more complete reports of the meta-analysis results. We have also detailed a diagnostic tool, which is essential to ensure the quality of the analysis.

Our method borrows heavily from the ALE method, but instead of a Gaussian function describing the uncertainty in location for each foci, a truncated Gaussian is used. Nevertheless, the ALE values computed by LocalALE are almost identical to those produced by GingerALE (figures 4 & 6). This is important since the ALE is the test statistic used for meta-analysis. Instead of testing for significantly high ALE in each voxel, we test only at the foci. Consequently complete experiments can be generated under the null hypothesis, stored, and processed; a task that would be very computationally demanding for a voxel-wise analysis. Computational hypothesis testing is performed by randomising the foci and iterating to generate a null distribution of ALE values. The randomisation is required to preserve the distribution of MA values in the observed data. Our method of randomising is different to that employed in the ALE and MKDA methods, but does preserve the MA on average as required (see figure 1). Clusters of significant foci form the results, and our algorithm can detect structure within the ALE that is important for complete reporting; rather than detecting connected voxel clusters, which can result in cluster merging (see table 3 and figure 6). Because we can efficiently store and process many realisations of the experimental data generated under the null, we can analyse the p-values and use them directly to control the type 1 statistical error. We can therefore control the FDR, despite tests not being independent. Most importantly we can count the number of clusters and control the directly relevant FCDR.

We have performed several experiments to compare results from LocalALE to the much used ALE algorithm [8] incorporated in GingerALE; keeping the processing options as close as possible.

We expected to see very few significant results from the randomised foci experiment; the numbers of false clusters found is depicted in figure 2. For this data BH-FDR and FDR control resulted in false clusters in a small fraction of experiments, while FCDR found none in 1000 experiments. GingerALE produced a median of 3 clusters per experiment, with an average size of 72 voxels (575 mm^3^). This is considerably larger than the suggested 45 voxel threshold suggested in [4]. These results suggest that, in the absence of consistent study data, the rate of false clusters is controlled best using FCDR, and that the many tests involved in a voxel-wise analysis may lead to increased false positive findings.

Diagnostic analysis (figure 3a and 3b) highlighted the importance of data checking. In the face perception experiment, of those with outlying overlap scores one study was found to be recorded incorrectly in the BrainMap.org; MNI coordinates recorded instead of Talairach. Another study tested a working and long term memory hypothesis, utilising face images, that may not have been functionally relevant. The overlap score revealed two outlying studies in the pain data, and these were removed from further analysis; one transformed coordinates from MNI to a different coordinate system, and while the other reported pain as a main effect it was combined with a cognitive task.

LocalALE produced similar results to GingerALE with the face perception data (figure 4). The use of a WB mask or GM mask made little overall difference to the results. There were several extra clusters declared significant by GingerALE but not LocalALE, and vice-versa using the FDR option in LocalALE (Table 1). All clusters found using FCDR were also found by FDR (LocalALE) and by GingerALE. Clusters found exclusively by GingerALE, were smaller than the average size found in the randomised foci experiment, and could be false positives. Those found significant by LocalALE but not GingerALE were only marginally significant at a FDR of 0.05, contained few experiments, and were not significant by FCDR; the use of FDR would in this case result in significant clusters even though a high proportion of those clusters are expected to be false.

To examine the control of false positive clusters in the presence of known true clusters, we generated randomised foci experiments from the face perception data, with the significant clusters found using FCDR preserved (foci involved in those clusters not randomised). For each of 1000 generated experiments we counted extra clusters beyond the original 9 (reported in table 1) using LocalALE. We also counted extra clusters in 100 of these experiments with GingerALE. The face perception data was ideal since we could employ cluster level control in GingerALE, and because any extra clusters were easily identifiable amongst the 9 true clusters. Figure 5 shows that FCDR performs best, as extra clusters are generated from the random foci in only 10% of the experiments. Contrast this with the results from GingerALE, which produced false clusters in over half of the experiments.

We tested our clustering algorithm by performing a meta-analysis of thermal pain stimulus in healthy volunteer subjects. Pain stimulus has been shown to produce extensive activation on fMRI. Figure 6 shows the ALE image of significant foci, and the clusters resulting from the analyses. LocalALE has found many more clusters than GingerALE (Table 3). Looking at the ALE images in figure 6, it is clear that there are distinct regions of high ALE, where the density of studies reporting foci is at a peak. Even though these regions merge, the magnitude of the ALE helps LocalALE to separate them into different clusters; along with the requirement that the foci must overlap to form clusters in LocalALE. By contrast GingerALE simply finds connected significant voxels, which in the pain analysis has merged, for example, left and right insula, and the left and right Thalamus, into just one cluster (see Table 3).

Clustering is clearly important for FCDR control, and it is essential for correct reporting of structures involved in the functional task. To test whether the results of FCDR were particularly sensitive to the details of the clustering scheme, we modified the algorithm such that it did not use the ALE to form clusters (see figure 7). While the clustering is quite different, the regions found to be significant by FCDR are very similar. This is likely because the clustering is modified both for the observed experiment and the null experiments. Therefore, the exact details of the algorithm used to detect clusters do not seem to substantially change the type 1 error control imposed by FCDR.

Testing only at the foci might limit the ability of LocalALE to resolve the shape of the significant clusters compared to voxel-wise analyses performed by other methods, since the foci are (relatively) sparse compared to the voxels. However, our results show that clusters tend to form in the same anatomical structures and even share shape features with those generated by the ALE algorithm. A Monte Carlo simulation is performed to estimate the p-values: the probability that the observed ALE is greater than, or equal to, the ALE values observed under the null hypothesis. To obtain converged estimates, many randomisations must be performed; the latest version of the ALE algorithm considers every possible randomisation, so the p-values are analytic and precise. We found that 10000 randomisations produced sufficiently converged p-values such that repeating the analysis, with different randomisations, did not change the results. A limitation of coordinate based meta-analysis in general is that it is unlikely to be able to reproduce exactly the results of pooled image based meta-analysis [12], [24]. To perform a meta-analysis closer to such schemes using the reported foci might require details, for example Z scores at the foci, that are not reported in a standard way. More importantly the null hypothesis, that the studies are not related by task/stimulus, is not strictly reflected in the null used to perform the Monte Carlo simulations used in CBMA. Nevertheless, a vast amount of coordinate based data is readily accessible, and CBMA is currently the accepted way to analyse it quantitatively.

### Conclusions

LocalALE tackles one of the major issues with the previously published CBMA algorithms, the multiple testing problem. As a direct consequence of our approach, we are able to control the intuitive False Cluster Discovery Rate, which relates directly to the results (clusters) of the meta-analysis; unlike schemes that control tests on voxels or foci. In comparison to the widely used ALE algorithm, LocalALE detects relatively few false positives. We demonstrated that checking the suitability of each study is essential, as mistakes are easily made with the data. We also detailed a clustering algorithm that provides a more complete report of significant results than the ALE algorithm. LocalALE is available to use freely as part of NeuROI (http://www.nottingham.ac.uk/scs/divisions/clinicalneurology/software/neuroi.aspx).

## Supporting Information

Appendix S1 (DOCX)

## References

[1] Talairach J, Tournoux P (1988) Co-planar stereotaxic atlas of the human brain. New York: Thieme.

[2] Costafreda SG (2009) Pooling fMRI data: meta-analysis, mega-analysis and multi-center studies. Frontiers in Neuroinformatics 3: 33–41. 19826498 10.3389/neuro.11.033.2009 PMC2759345

[3] Chein JM, Fissell K, Jacobs S, Fiez JA (2002) Functional heterogeneity within Broca's area during verbal working memory. Physiol Behav 77: 635–639. 12527011 10.1016/s0031-9384(02)00899-5

[4] Eickhoff SB, Bzdok D, Laird AR, Kurth F, Fox PT (2012) Activation likelihood estimation meta-analysis revisited. Neuroimage 59: 2349–2361. 21963913 10.1016/j.neuroimage.2011.09.017 PMC3254820

[5] Eickhoff SB, Laird AR, Grefkes C, Wang LE, Zilles K, et al (2009) Coordinate-based activation likelihood estimation meta-analysis of neuroimaging data: a random-effects approach based on empirical estimates of spatial uncertainty. Hum Brain Mapp 30: 2907–2926. 19172646 10.1002/hbm.20718 PMC2872071

[6] Laird AR, Fox PM, Price CJ, Glahn DC, Uecker AM, et al (2005) ALE meta-analysis: controlling the false discovery rate and performing statistical contrasts. Hum Brain Mapp 25: 155–164. 15846811 10.1002/hbm.20136 PMC6871747

[7] Laird AR, McMillan KM, Lancaster JL, Kochunov P, Turkeltaub PE, et al (2005) A comparison of label-based review and ALE meta-analysis in the Stroop task. Hum Brain Mapp 25: 6–21. 15846823 10.1002/hbm.20129 PMC6871676

[8] Turkeltaub PE, Eickhoff SB, Laird AR, Fox M, Wiener M, et al (2012) Minimizing within-experiment and within-group effects in Activation Likelihood Estimation meta-analyses. Hum Brain Mapp 33: 1–13. 21305667 10.1002/hbm.21186 PMC4791073

[9] Wager TD, Lindquist M, Kaplan L (2007) Meta-analysis of functional neuroimaging data: current and future directions. Soc Cogn Affect Neurosci 2: 150–158. 18985131 10.1093/scan/nsm015 PMC2555451

[10] Wager TD, Phan KL, Liberzon I, Taylor SF (2003) Valence, gender, and lateralization of functional brain anatomy in emotion: a meta-analysis of findings from neuroimaging. Neuroimage 19: 513–531. 12880784 10.1016/s1053-8119(03)00078-8

[11] Radua J, Mataix-Cols D (2009) Voxel-wise meta-analysis of grey matter changes in obsessive-compulsive disorder. Br J Psychiatry 195: 393–402. 19880927 10.1192/bjp.bp.108.055046

[12] Radua J, Mataix-Cols D, Phillips ML, El-Hage W, Kronhaus DM, et al (2012) A new meta-analytic method for neuroimaging studies that combines reported peak coordinates and statistical parametric maps. Eur Psychiatry 27: 605–611. 21658917 10.1016/j.eurpsy.2011.04.001

[13] Turkeltaub PE, Eden GF, Jones KM, Zeffiro TA (2002) Meta-analysis of the functional neuroanatomy of single-word reading: method and validation. Neuroimage 16: 765–780. 12169260 10.1006/nimg.2002.1131

[14] Benjamini Y, Hochberg Y (1995) Controlling the False Discovery Rate: A Practical and Powerful Approach to Multiple Testing. Journal of the Royal Statistical Society Series B (Methodological) 57: 289–300.

[15] Chumbley JR, Friston KJ (2009) False discovery rate revisited: FDR and topological inference using Gaussian random fields. Neuroimage 44: 62–70. 18603449 10.1016/j.neuroimage.2008.05.021

[16] Heller R, Stanley D, Yekutieli D, Rubin N, Benjamini Y (2006) Cluster-based analysis of FMRI data. Neuroimage 33: 599–608. 16952467 10.1016/j.neuroimage.2006.04.233

[17] Benjamini Y, Hochberg Y (2000) On the Adaptive Control of the False Discovery Rate in Multiple Testing With Independent Statistics. Journal of Educational and Behavioral Statistics 25: 60–83.

[18] Fox PT, Laird AR, Fox SP, Fox PM, Uecker AM, et al (2005) BrainMap taxonomy of experimental design: description and evaluation. Hum Brain Mapp 25: 185–198. 15846810 10.1002/hbm.20141 PMC6871758

[19] Fox PT, Lancaster JL (2002) Opinion: Mapping context and content: the BrainMap model. Nat Rev Neurosci 3: 319–321. 11967563 10.1038/nrn789

[20] Laird AR, Lancaster JL, Fox PT (2005) BrainMap: the social evolution of a human brain mapping database. Neuroinformatics 3: 65–78. 15897617 10.1385/ni:3:1:065

[21] Dijkstra EW (1959) A note on two problems in connexion with graphs. Numerische Mathematik 1: 269–271.

[22] Platek SM, Loughead JW, Gur RC, Busch S, Ruparel K, et al (2006) Neural substrates for functionally discriminating self-face from personally familiar faces. Hum Brain Mapp 27: 91–98. 16035037 10.1002/hbm.20168 PMC6871291

[23] Braver TS, Barch DM, Kelley WM, Buckner RL, Cohen NJ, et al (2001) Direct Comparison of Prefrontal Cortex Regions Engaged by Working and Long-Term Memory Tasks. Neuroimage 14: 48–59. 11525336 10.1006/nimg.2001.0791

[24] Salimi-Khorshidi G, Smith SM, Keltner JR, Wager TD, Nichols TE (2009) Meta-analysis of neuroimaging data: a comparison of image-based and coordinate-based pooling of studies. Neuroimage 45: 810–823. 19166944 10.1016/j.neuroimage.2008.12.039

