# Supplementary material for: Coordinate Based Meta-Analysis of Functional Neuroimaging Data; False Discovery Control and Diagnostics
Source: PLoS One. 2013 Jul 29;8(7):e70143. doi: 10.1371/journal.pone.0070143 (PMC3726528; doi:10.1371/journal.pone.0070143)
Supplement: Appendix S1 — (DOCX) [file pone.0070143.s001.docx]

# Appendix S1

**Meta-analysis algorithm**

**Function Meta**(**r**)

- Let **r** contain elements r*_ij_*; the *i*th focus in the *j*th study

//*This algorithm performs meta-analysis on the experiment contained in* **r**

BEGIN

//*generate 2000 null experiments; realisations of the experiment under the null hypothesis*

for 1≤ *k* ≤2000 **r0***_k_* = **Randomise**(**r**)

Let **r0** have elements **r0***_k_*

//*generate the cumulative distribution function of ALE values under the null hypothesis; use 10000 randomisations*

for 1≤ *k* ≤10000

**r**’ = **Randomise**(**r**)

**A** = **ALE**(**r**’)

Add ALE values **A** to the distribution function of ALE: df(*A*)

endfor

From df(*A*) generate the cumulative distribution cdf(*A*)

//*Compute the p-values for the observed foci*

**P** = **Pvalues**(cdf, **r**)

//*Compute the p-values for the 2000 null experiments*

for 1≤ *k* ≤2000 **P0***_k_* = **Pvalues**(cdf, **r0***_k_*)

Let **P0** have elements **P0***_k_*

//*Compute the test level* α_FDR_ *that controls the Type 1 error using FDR*

α_FDR_ = **FDR**(**P**, **P0**, 0.05)

*//Foci with p-values ≤* α_FDR_ *are significant controlling for FDR at a level of 0.05*

//*Compute the test level* α_FCDR_ *that controls the Type 1 error using FCDR*

α_FCDR_ = **FCDR**(**P**, **r**, **P0**, **r0**, 0.05)

*//Foci with p-values ≤* α_FCDR_ *are significant controlling for FCDR at a level of 0.05*

END

**Function Randomise**(**r**): return a randomisation of the foci preserving within study clusters of foci, as detailed in the methods, and avoiding overlapping foci within study

**Function ALE**(**r**): Compute, and return, the ALE for each focus in **r** using equations 1 & 2 in the methods

**Function Pvalues**(cdf, **r**): Compute the ALE value *A_ij_* for each focus r*_ij_* in **r** and let the p-value P*_ij_ =* (1-cdf(*A_ij_*)). Return **P** containing elements P*_ij_*

**Function FDR**(**P**, **P0**, γ):

For level α:

Estimate the number of rejections in the observed experiment: N = count(P*_ij_* ≤ α)

Estimate the mean number of rejections in the null experiments: N0 = count(P0*_ijk_* ≤ α)/2000

Maximise α such that N0/N ≤ γ

Return α

**Function FCDR**(**P**, **r**, **P0**, **r0**, γ):

For level α:

Estimate the number of clusters in the observed experiment: N, using the clustering algorithm for foci **r** for significant foci at level α

Estimate the mean number of clusters in the null experiments: N0, using the clustering algorithm for foci **r0*_k_*** for significant foci at level α

Maximise α such that N0/N ≤ γ

Return α

**Cluster finding algorithm**

To assign foci to clusters Dijkstra's shortest path algorithm is utilised. We begin by finding peaks in the ALE, then assigning the nearby foci to the cluster associated with that peak. Cluster finding is particularly important in CBMA since it is the clusters that form the results. In LocalALE they are of particular importance since they are used to control the false positive results.

Clusters start at a peak in the ALE value. They grow to include significant foci (p-value ≤ α) that overlap each other, and foci that overlap with the significant foci. A cluster is constrained such that the ALE value should be reducing (but not strictly reducing) away from the peak. Where clusters merge, such that foci could be assigned to one of several clusters, they are assigned to the closest by the shortest path algorithm.

- Let r*_i_* be the location of the *i*th focus considered for clustering; foci considered are those declared significant by the meta analysis, or foci that are located <2.8σ from a significant focus
- Let *A_i_* be the ALE at focus r*_i_*
- Let *C_i_* be the cluster focus r*_i_* is assigned to; initialised to zero
- Let δ*_ij_* be the distance separating focus r*_i_* from r*_j_*; δ*_ij_*= δ*_ji_*=|r*_i_ –* r*_j_*|
- Let *d_i_* be the distance from focus *i* from the nearest cluster peak; initialise to ∞
- Let counter CLUSTER=1

The clustering algorithm uses a heap data structure to make it efficient. In this case foci are entered into the heap, and the data structure sorted such that the focus with the smallest *d_i_* is always at the top, and those foci with the largest *d_i_* always at the bottom. Operations that can be performed on the heap are: **insert**, **remove**, and **update**. These operations are performed such that the heap remains sorted at all times.

1. Find the focus r*_i_* with the largest *A_i_* (a peak) and with *C_i_*=0, and set *C_i_*=CLUSTER
2. **Insert** r*_i_* onto the heap, and set *d_i_*=0
3. From all foci currently in the heap, **remove** that with the smallest *d_i_*: r*_k_*
4. For all r*_i_* with δ*_ik_* < 2.8σ AND *A_i_* ≤ *A_k_* AND *d_k_* + δ*_ik_* < *d_i_* do:

Let *d_i_* = *d_k_* + δ*_ik_*

Let *C_i_* = *C_k_*

If r*_i_* not already in the heap, **insert** it now

Otherwise **update** r*_i_* in the heap

1. Repeat from 3 while there are still foci in the heap structure
2. Let CLUSTER = CLUSTER+1
3. Repeat from 1 while there are still foci to assign to clusters
